# Degradation Characteristics of Reed-Based PBAT Mulch and Their Effects on Plant Growth and Soil Properties

**DOI:** 10.3390/ma18071477

**Published:** 2025-03-26

**Authors:** Yipeng Wang, Qiuxia Zhang, Yinghao Huang, Jia Xu, Jixing Xie

**Affiliations:** 1 School of Eco-Environment, Hebei University, Baoding 071000, China; 18231307110@163.com (Y.W.); 15234529298@163.com (Q.Z.); 13739619873@163.com (Y.H.); 2 Xiong’an Institute of Innovation, Baoding 071700, China

**Keywords:** poly(butylene adipate-co-terephthalate), reed, mulch film, soil environment, plant growth

## Abstract

Poly (butylene adipate-co-terephthalate) (PBAT) and PBAT/reed fiber (RF) mulch films were prepared. The molecular structural changes and surface morphological evolution during the degradation process were systematically characterized using Fourier-transform infrared spectroscopy (FTIR) and scanning electron microscopy (SEM). The prepared PBAT/RF mulch film biodegradation rate reached 90.43% within 91 days under controlled composting conditions, which was 9.52% higher than a pure PBAT mulch film. The effects of adding PBAT and PBAT/RF microplastics on soil properties and soybean physiological indicators were dynamic. The study demonstrated that the incorporation of 5% PBAT/RF mulch film fragments into soil led to a 5.1% reduction in soil pH and a 17.2% increase in soluble organic carbon content. While the effects of 5% PBAT/RF on soil urease and neutral phosphatase activities were non-significant, sucrase activity decreased by 7.4% and catalase activity was reduced to 0.38 U/g. Additionally, the addition of 5% PBAT/RF resulted in a soybean germination rate of 93.74%, which was 4.0% higher than that observed in the group treated with 5% PBAT alone. The experimental data revealed a 7.2% reduction in leaf chlorophyll content, with concomitant growth inhibition in the soybean seedlings. The study demonstrated that the PBAT/RF composite film achieved 89% biodegradation within 180 days under field conditions, effectively mitigating post-application effects on agroecosystems compared to conventional polyethylene mulch.

## 1. Introduction

A mulch film covering can improve the microclimate of soil surface by increasing the reflectivity of sunlight and the airflow resistance [1]. Thus, it increases the net photosynthetic rate of crop leaves to achieve increased crop yields and efficiency [2]. Mulch filming is particularly important given the increased demand for food due to global population growth [3,4]. In a past study, the growth of soybean seedlings was inhibited, and a 7.2% decrease in leaf chlorophyll content was observed. The study’s findings suggest that PBAT/RF mulch demonstrates enhanced degradation properties, effectively minimizing its detrimental effects on both plants and soil following its use [5]. Therefore, the development of biodegradable alternatives and a reduction in the use of traditional plastic mulch film are of great significance for the protection of the soil environment and the maintenance of ecological balance [6]. Polybutylene terephthalate (PBAT) is an aliphatic aromatic biodegradable polyester [7,8]. As a biodegradable material, PBAT mulch film has broad application prospects. Its degradation characteristics and its impact on the soil environment have attracted great attention [9,10].

Biodegradable mulch films release organic matter and debris during degradation, which affects the physical properties of soil. The infiltration of these materials changes the texture and structure of soil, affecting aeration, water retention, and resistance to wind erosion [11,12]. The degradation products of biodegradable films also affect the chemical properties of soil, including pH, ion exchange capacity, and nutrient content. Such alterations can affect the ability of plants to absorb nutrients from soil, thereby affecting normal plant growth and development. In addition, biodegradable films release micron and nanoscale plastics, additives, monomers, and other intermediates as they are degraded by soil microorganisms [13,14]. Repeated or prolonged use of biodegradable mulch film may lead to the accumulation of these substances in soil, which could adversely affect soil quality, soil organisms, and crop growth [15,16].

The impact of degradable plastics on plant growth remains uncertain. Corn biomass and chlorophyll content decreased when they were grown in soil containing 10% (*w*/*w*) polylactic acid (PLA) fragments [17]. Additionally, Lian et al. demonstrated that PLA significantly inhibited soybean root growth and disrupted the antioxidant system in the leaves [18]. It was also found that 10% poly (3-hydroxybutyrate-co-3-hydroxyvalerate) (PHBV) caused the death of wheat plants after 25 days [19]. Muroi et al. buried 1% (*w*/*w*) PBAT debris in soil, and after 7 months, no effects on the growth of Brussels sprouts were observed [20]. Sforzini et al. found that both monocotyledonous and dicotyledonous plants planted in soils containing polylactic acid/PBAT mulch film debris were not affected [21]. Degradable plastics produce biodegradable MPs in the environment, and these are currently understudied [22,23]. Whether or not PBAT biodegradable mulch film is also detrimental to plants and microbial systems is an open question [24].

In recent years, research has shown that blending natural plant fibers with PBAT can enhance the degradation rate of composite materials [25]. However, further investigation into the degradation mechanisms of these materials is necessary to expand the options for the application and development of biodegradable mulch. This experiment also measured and analyzed the effects of PBAT and PBAT/RF microplastics on soil properties and soybean growth. This study demonstrates that the incorporation of plant fibers significantly mitigates the impact of PBAT films on both soil ecosystems and plant growth. The experimental data obtained provide crucial insights for the scientific assessment of ecological safety while offering theoretical foundations for the application and optimization of biodegradable mulch films. These findings hold substantial implications for advancing green and low-carbon agricultural practices, contributing to sustainable development initiatives.

## 2. Materials and Methods

### 2.1. Materials

PBAT (TH801T) was purchased from Xinjiang Blue Ridge Tunhe Polyester Co., Ltd. (Changji, China). Reeds were collected from the shore of a lake (Baiyangdian, Baoding, China). Other chemical reagents were obtained from Sinopharm Chemical Reagent (Shanghai, China).

### 2.2. Preparation of the Sample Mulch Film

The reed fibers (RF) were dried, milled, and passed through a 500-mesh sieve. The PBAT pellets and RF were dried at 60 °C for more than 24 h before extrusion. The PBAT and RF were weighed at a ratio of 8:2, and the material was shaken uniformly through a mixer. The mixer was then shaken uniformly for 20 min. The single-screw extruder was equipped with a screw diameter of 20 mm and an L/D ratio of 40. The screw was a co-rotating meshing type where different forms of threaded units were assembled to form the threaded section. The thickness tester (PY-H606E, Shenzhen Puyun Electronic Co., Ltd., Shenzhen, China) was used to measure the film thickness according to the detection methods specified in the GB/T 6672-2001 [26]. The film thickness was measured to be 20–25 mm. The twin screw extruder was set at 45 rpm with a temperature of 140–155 °C to blend the PBAT and RF mixture. The temperature range was 140–155 °C. For comparison, pure PBAT was extruded and pelletized under the same conditions. We dried the pellets for more than 24 h in an oven set at 60 °C. The blown PBAT and PBAT/RF films were extruded on a single-screw extruder set at 300 rpm. The temperature was 150 °C. The films were named PBAT and PBAT/RF and were 30 μm thick. The cut sizes of the PBAT and PBAT/RF films were 3 mm × 3 mm × 0.035 mm.

### 2.3. Compost Degradation Test

For the compost biodegradation, mature compost was obtained from the Beijing Nangong composting plant. This compost material was primarily derived from urban domestic waste and was classified as a multi-purpose compost. As shown in Table 1, five compost reactors were used, with two control groups for each composition. The first set of containers contained 300 g of compost as a blank control. The second set of containers contained a mixture of 300 g of compost and 50 g of cellulose powder as control sample (a). The third set of containers contained a mixture of 300 g of compost and 50 g of reed fibers (a composite with PBAT/RF) as control sample (b). The fourth set of containers contained 300 g of compost and 50 g of pure PBAT. The fifth set of containers contained 300 g of compost and 50 g of PBAT/RF. The reactors were placed at 58 ± 2 °C and 45–55% humidity for 90 days. All reactors were designed according to GB/T19277-2011 [27]. The compost humidity was adjusted every 3 days, and the materials were agitated to prevent clumping, along with stirring to prevent caking. The CO_2_ generated during the biodegradation of the compost was trapped in a 1 M NaOH solution. Samples were taken every 7 days and analyzed by standard sodium hydroxide titration to determine the volatilized CO_2_ in the reactor. After titration, we replaced the NaOH solution and added the newly configured NaOH solution to the absorption vessel.

The biodegradation rate was calculated as follows:

① The theoretical release of the CO_2_ was calculated as shown in Equation (1):

(1) $${ThCO}_{2}=M_{TOT}\times C_{TOT}\times 44/12,$$

where M*_TOT_* is the mass of the total dry solids added to the compost container at the start of the test (g) and C*_TOT_* is the ratio of the total organic carbon to the total dry solids in the test material.

② The percentage of biodegradation was based on the cumulative amount of CO_2_ released, and the rate of biodegradation (*D_t_*) was evaluated by using Equation (2):

(2) $$D_{t}\left(\%\right)=\left[{\left({CO}_{2}\right)}_{T}-{\left({CO}_{2}\right)}_{B}\right]/Th{CO}_{2}\times 100,$$

where (CO_2_)*_T_* represents the amount of CO_2_ produced in the sample, (CO_2_)*_B_* represents the amount of CO_2_ produced in the blank control, and ThCO_2_ represents the theoretical amount of CO_2_ that the sample mulch film could produce in each composting reactor. This was determined by using a total organic carbon analyzer (TOC, TOC-2000, Shanghai Metash Instruments Co., Ltd., Shanghai, China) and then performing the calculation.

#### 2.3.1. FTIR Analysis

The samples were tested by Fourier-transform infrared spectroscopy (FTIR) before and after compost degradation. The attenuated total reflection (ATR) mode was used. For all spectra, the resolution was 4 cm^−1^ and the wavelength range was 4000 to 500 cm^−1^.

#### 2.3.2. SEM Morphology

Each specimen before and after compost degradation was pressed and sprayed with gold, and the microstructure of the film surface was observed using a scanning electron microscope (SEM, TM4000PLUS, Hitachi, Tokyo, Japan) at an accelerating voltage of 15 kV.

#### 2.3.3. Transmittance of the Films

The optical properties of the PBAT and PBAT/RF composite films were measured using a calibrated light transmission meter. Before measurement, the instrument was preheated for 30 min to ensure stability. The film samples, cut into 3 cm × 5 cm rectangles, were placed on the instrument’s clamping plate for analysis. To improve the reliability and account for material variation, five measurements were taken at different spots on each film. The final transmittance value for each sample was the average of these five readings, ensuring accuracy and consistency in the results.

#### 2.3.4. Elongation at the Break of the Material

The tensile properties were measured using an electronic universal testing machine (Shenzhen Sansi Zongheng Technology Co., Ltd., Shenzhen, China) following GB/T 1040.2-2006 [28]. Dumbbell-shaped specimens (75 × 4 × 2 mm) were tested at 25 °C with a crosshead speed of 10 mm min^−1^. Five replicate tests were performed for each sample, and the results were averaged to ensure data reliability.

### 2.4. Plant Cultivation

#### 2.4.1. Effects of the PBAT and PBAT/RF Mulch Films on the Soil Properties

The study used soil samples collected from a depth of 10–30 cm in an organically fertilized farmland located in Anxin County, Xiong’an New Area. The key properties of the soil were determined to be as follows: a pH of 6.78, water content of 23.5%, soluble organic carbon content of 107.33 mg kg^−1^, and total nitrogen content of 641.46 mg/kg. Each 8 kg of soil aliquot was amended with the cryomilled PBAT and PBAT/RF fragments (particle size of ≤0.5 mm) at concentrations of 0.1%, 0.5%, 1%, and 5% (*w*/*w*). The soil-polymer mixtures were homogenized using a mechanical mixer and subsequently transferred to cultivation pots. All treatments were performed in triplicate, including an unamended control (CK) to establish baseline conditions.

(1) Determination of the basic physical and chemical properties of the soil

The soil pH determination was performed as follows: a pH meter method was used to determine a water–soil ratio of 2.5:1 (*w*/*w*). The determination of the soil’s dissolved organic matter (DOM) content was conducted as follows: 10.00 g of soil samples were extracted from potting soil into 500 cm^3^ triangular flasks. Subsequently, 200 cm^3^ of deionized water was added for leaching, followed by centrifugation at 4000 rpm for 10 min after 30 min of incubation and agitation at 25 °C. The resulting supernatant was then extracted and filtered through a 0.45 μm micropore filter membrane, yielding the soil’s DOM. This filtrate was designated as the soil DOM extract, and the soil’s DOM content was quantified using a total organic carbon (TOC) meter. The total soil nitrogen was determined with an SKD-1000 Automatic Kjeldahl Nitrogen Analyzer (Peiou Analysis Instrument Co., Ltd., Shanghai, China).

(2) Measurement of the soil’s enzyme activity

The enzyme activity was determined by the kit method (Solarbio Science & Technology Co., Ltd., Beijing, China). The soil urease activity was assessed by a sodium phenol-sodium hypochlorite colorimetric assay, the soil catalase activity was analyzed by a UV colorimetric assay, the soil phosphatase activity was determined by a disodium phosphate colorimetric assay, and the soil sucrase activity was determined by a 3,5-dinitrosalicylic acid colorimetric assay. The sucrase, catalase, soil urease, and phosphatase levels were colorimetrically measured at 508 nm, 415 nm, 578 nm, and 660 nm, respectively, on a TU-1810 UV spectrophotometer (Beijing Purkinje General Instrument Co., Ltd., Beijing, China).

#### 2.4.2. Effect of the PBAT and PBAT/RF Mulch Films on Plant Growth

(1) Determination of the germination rate

A germination rate test was conducted in a sunlit greenhouse with an average indoor daytime temperature of 22.6 ± 0.7 °C. Throughout the soybean seed germination process, the daily count of germinating soybean seeds over 7 days was meticulously documented. The soybean seed germination rate was determined by calculating the percentage of seeds that successfully germinated within 7 days out of the total seeds tested.

(2) Measurement of plant height, root length, and total biomass

The soybean plants were cultivated under controlled greenhouse conditions for 21 days, after which the seedlings were carefully extracted from their pots. The seedlings were gently separated from the growth medium, and any residual soil particles adhering to the root systems were removed. They were then thoroughly rinsed with tap water, followed by three successive rinses with deionized water. Finally, the seedlings were dried to remove the surface moisture before further analysis. The root lengths and plant heights of the seedlings were then measured using a ruler, and the weights of the soybean seedlings were determined using an electronic balance.

(3) Determination of the chlorophyll content

Five soybean seedlings were randomly chosen from each potted plant, and one leaf was selected from each seedling for chlorophyll measurement. A SPAD-502 Plus portable chlorophyll meter (Konica Minolta Optics, Osaka, Japan) was used to determine the chlorophyll index of the soybean seedling leaves. Three regions of each leaf were assessed and averaged. Subsequently, the mean value of the leaves from the 5 seedlings was calculated as the chlorophyll content of the sample plant in the potted plant.

The experimental results were expressed as means ± standard deviations and were processed by Excel 2022 (Microsoft, Redmond, WA, USA), and the plotting and image information were processed by Origin 2019 (OriginLab Co., Northampton, MA, USA) and ImageJ 1.53t (National Institutes of Health, Bethesda, MD, USA).

## 3. Results and Discussion

### 3.1. Mulch Properties

The elongations at break of the PBAT and PBAT/RF were 539.11% and 410.26%, respectively, and the transmittances were 52.5% and 12.6%, respectively. The addition of reed fibers decreased the elongation at break and transmittance of the PBAT/RF. The agglomeration behavior of the reed fibers resulted in poor compatibility between the reed fibers and the PBAT, which made the PBAT/RF films more susceptible to fracture and led to an increase in the scattering of light from the film surface.

### 3.2. Compost Degradation

#### 3.2.1. Composting Biodegradation

**Figure 1:**
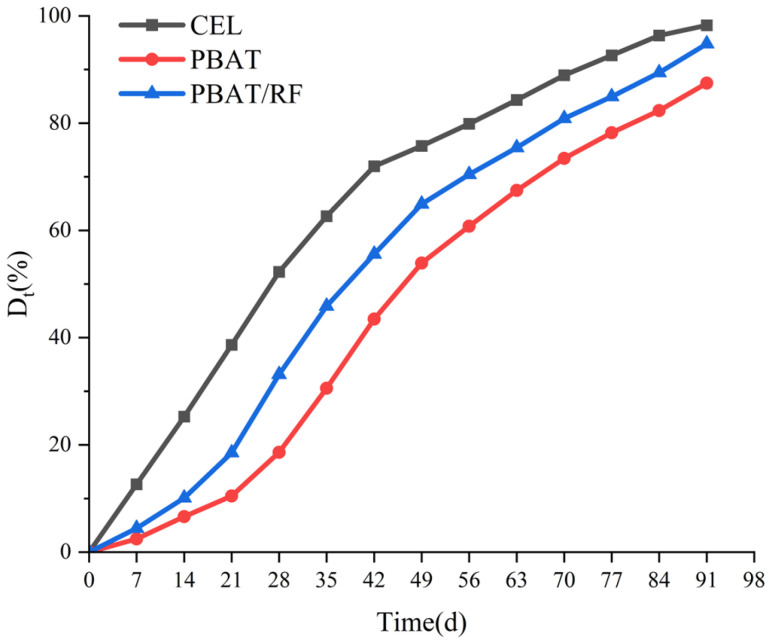
The rate of biodegradation (D_t_) for the CEL, PBAT, and PBAT/RF composites under composting conditions.

The biodegradation curve (Figure 1) revealed distinct degradation patterns for the cellulose (CEL) and PBAT. The cellulose exhibited no lag phase, with its biodegradation rate increasing rapidly from 0 to 49 days before reaching a plateau. By the end of the test period, the cellulose achieved a biodegradation rate of 98.23%, with approximately 70% degradation occurring within the first 42 days. These results demonstrated the effectiveness of the experimental system established in this study. In contrast, the PBAT displayed a short lag phase during the initial 21 days of degradation, which could be attributed to the time required for microbial adhesion and colonization on the material surface. This initial phase is critical as hydrolysis predominantly governs the early stages of degradation. The degradation rate started at 14 days and ended at 91 days, with a steady increase, and it finally reached 94.78%. This was similar to the degradation rate of a degradable biofilm consisting of a mixture of starch and PBAT under composting conditions of approximately 85–95% as prepared by Sintim et al. [29]. Microorganisms colonizing the sample surface secreted a range of depolymerization enzymes, including lipase, protease, and cellulase. These enzymes catalyzed the breakdown of ester bonds in the PBAT molecular chains, resulting in the formation of small molecules or oligomers. These degradation products underwent further hydrolysis and enzymatic reactions, ultimately being metabolized by the microorganisms into CO_2_ and H_2_O. Compared with the pure PBAT, the biodegradation delay period of the PBAT/RF composites supplemented with 20% reed fibers was reduced by 27.5%, and the biodegradation degree was increased by 7.33% at 91 days, which was in agreement with the findings of Giri et al. where the degradation of PBAT composites was improved [30].

**Figure 2:**
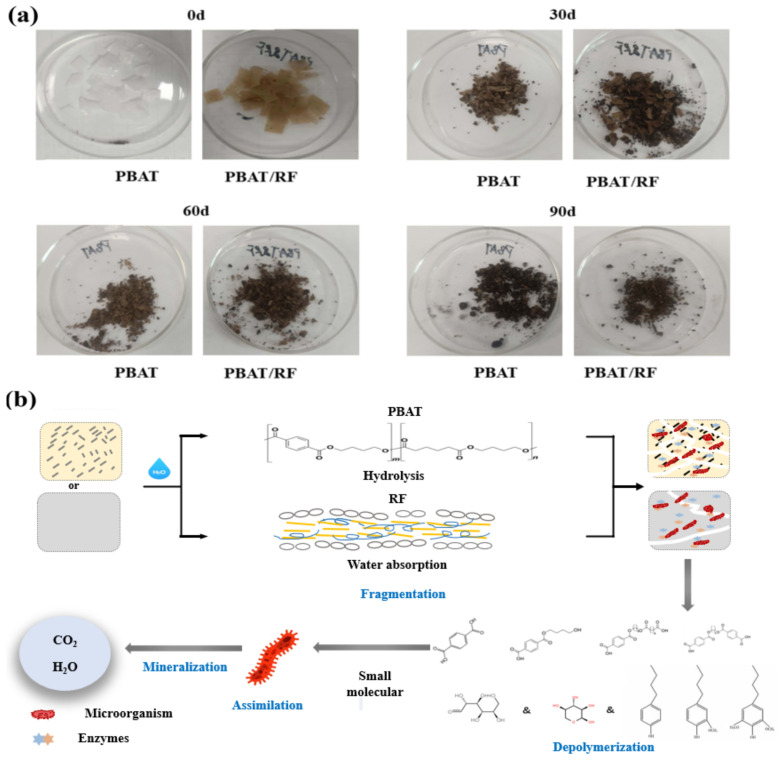
Photographs of the PBAT and PBAT/RF composites before and after composting degradation for 30, 60, and 90 days (**a**). PBAT and PBAT/RF biodegradation mechanism diagram (**b**).

As can be seen in Figure 2a, during the degradation process, the material continued to become smaller with increasing time, and at the end of the experiment, most of the samples had disappeared. The reed fibers made the composite more hydrophilic, which affected the rate of water diffusion during degradation, and the enzymes released by the microorganisms could break it down into sugars for direct use and provide more adhesion points for the microorganisms to improve the accessibility of the enzymes. The biodegradation processes and mechanisms of the PBAT and PBAT/RF are shown in Figure 2b. The PBAT absorbed water molecules in the soil for hydrolysis so that large fragments of PBAT were cleaved into small fragments, which were depolymerized by enzymes in the soil to form small molecules, and finally, they mineralized into carbon dioxide and water by assimilation.

#### 3.2.2. Infrared Analysis of the PBAT and PBAT/RF Films

**Figure 3:**
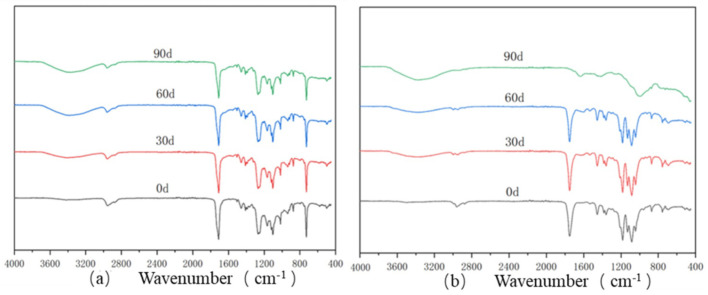
Infrared spectral analysis of the PBAT (**a**) and PBAT/RF (**b**) composites at days 0, 30, 60, and 90 of composting degradation.

The infrared spectral changes in the PBAT and PBAT/RF composites during the degradation process are shown in Figure 3. The FTIR spectral analysis revealed that the primary characteristic absorption peaks of the PBAT and PBAT/RF composites remained largely unchanged following 90 days of composting degradation. However, a notable intensification was observed in the -OH stretching vibration band at 3390 cm^−1^, which was indicative of an increased hydroxyl group concentration resulting from the degradation process. In addition, the -C=O peak at 1712 cm^−1^ showed a decreasing absorption intensity with time, which proved that the ester bond was continuously broken with the microbial activity. This was consistent with the findings of Xie et al., where the addition of reed fibers enhanced the degradation properties of the materials [31].

#### 3.2.3. Electron Microscopic Analysis of the PBAT and PBAT/RF Mulch Films

**Figure 4:**
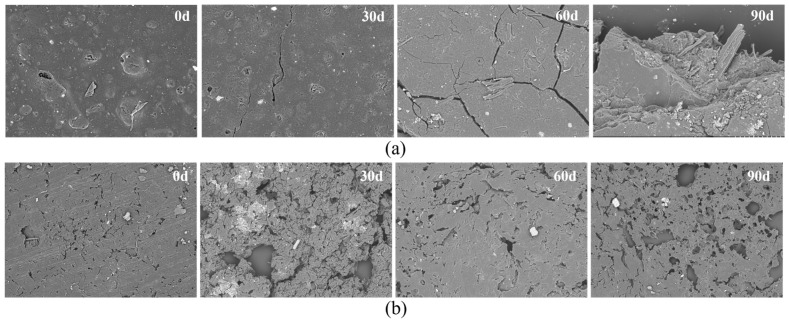
SEM micrographs of the PBAT (**a**) and PBAT/RF (**b**) composites at days 0, 30, 60, and 90 of composting degradation.

The SEM images in Figure 4 show the changes in the PBAT and PBAT/RF materials during compost biodegradation, respectively. On the 30th day, the surfaces of the PBAT film samples showed tiny cracks, which gradually increased with the degradation time, and air holes also appeared. Ninety days later, the surfaces of the PBAT films showed large areas of chipping, which indicated that the microbial erosion was gradually more serious. The PBAT/RF composite exhibited accelerated surface erosion compared to the pristine PBAT under identical experimental conditions, demonstrating enhanced biodegradability. Progressive detachment of the reed fibers from the PBAT matrix resulted in the formation of microcracks and cavities of varying dimensions. This phenomenon was attributed to the hydrophilic functional groups within the reed fibers, which exhibited an enhanced affinity for microbial colonization and water absorption in the compost environments. These interactions promoted the accelerated hydrolysis of the polymer matrix, thereby amplifying the composite’s biodegradation kinetics.

### 3.3. Effects of the PBAT and PBAT/RF Mulch Films on the Soil’s Properties

#### 3.3.1. PBAT and PBAT/RF Mulch Films’ Effects on pH

**Figure 5:**
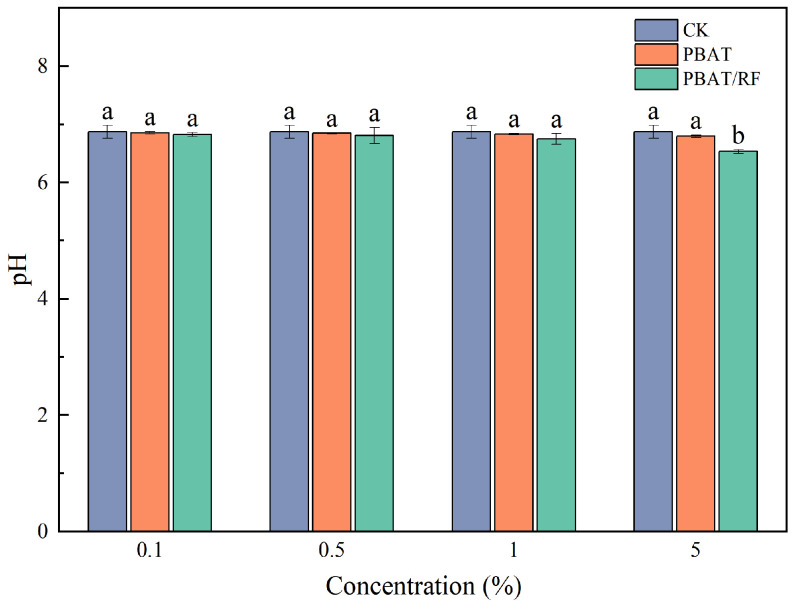
Effects of adding different concentrations of PBAT and PBAT/RF on soil pH. The different lowercase letters indicate significant differences across the various treatments at the same growth stage (*p* < 0.05), and the error bars represent the standard deviations.

The pH levels of the soil in the potted plants under various treatments are illustrated in Figure 5. In comparison to the control group (CK), no significant differences in soil pH were observed in the PBAT treatment group. Similarly, the soil pH in the PBAT/RF treatment group at or below a 1% concentration did not show significant variances. However, at a 5% concentration, a slight decrease in soil pH was noted. This phenomenon was consistent with Wang’s findings [32]. This reduction could potentially be attributed to the generation of adipic acid through the degradation of the PBAT/RF mulch film remnants. It is plausible that the utilization of the reed fibers by soil bacteria as a nutrient metabolism also contributed to the observed acidity.

#### 3.3.2. PBAT and PBAT/RF Mulch Films’ Effects on DOM

**Figure 6:**
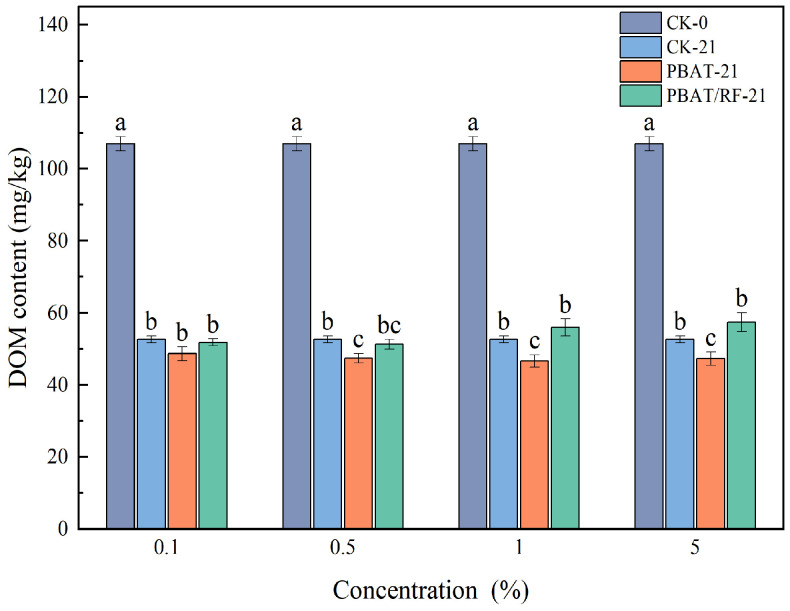
DOM of the soil under the different treatments. The different lowercase letters indicate significant differences across the various treatments at the same growth stage (*p* < 0.05), and the error bars represent the standard deviations.

The soluble organic carbon contents in the soil before cultivation and at harvest are depicted in Figure 6. Across all control groups, there was a decrease in the dissolved organic matter (DOM) content in the soil. This decline primarily resulted from the substantial nutrient uptake by the soybeans during the initial growth stages, leading to a depletion of soil nutrients. In comparison to the control (blank), there was no significant difference in the soil DOM content observed with varying PBAT concentrations. However, the results for a 5% PBAT/RF treatment were consistent with Ma’s study [33]. The soil DOM content increased in contrast to the blank soil during the corresponding period. This rise, reaching 60.16 mg kg^−1^, could be attributed to the presence of water-soluble, low-molecular-weight polymers generated through the degradation of the PBAT/RF mulch film. These polymers contributed to the increase in soil DOM content.

#### 3.3.3. Effects of the PBAT and PBAT/RF Mulch Films on the Soil’s Total Nitrogen Contents

**Figure 7:**
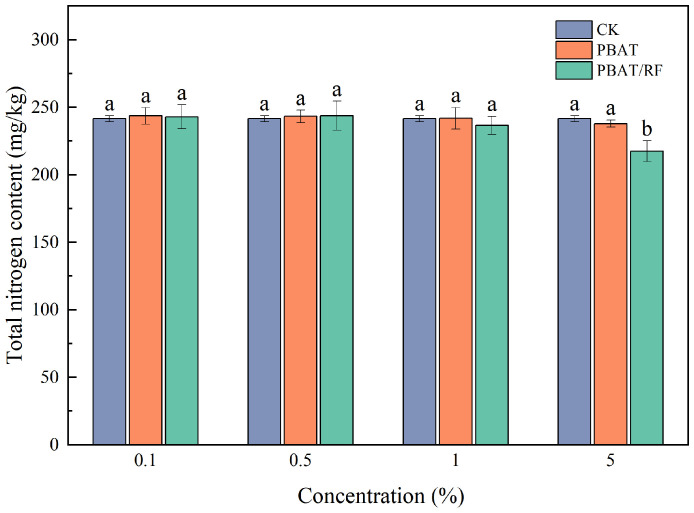
Total nitrogen contents of the soil under the different treatments. The different lowercase letters indicate significant differences across the various treatments at the same growth stage (*p* < 0.05), and the error bars represent the standard deviations.

Figure 7 demonstrates that the residual PBAT and PBAT/RF mulch films induced no statistically detectable alterations in soil total nitrogen contents across all concentration gradients. As these polymeric materials were predominantly composed of carbonaceous constituents, their mineralization processes negligibly influenced the soil nitrogen dynamics. The limited degradation timeframes further constrained their capacity to interfere with the plants’ nitrogen assimilation pathways.

#### 3.3.4. Effects of the PBAT and PBAT/RF Mulch Films on the Soil’s Enzyme Activities

**Figure 8:**
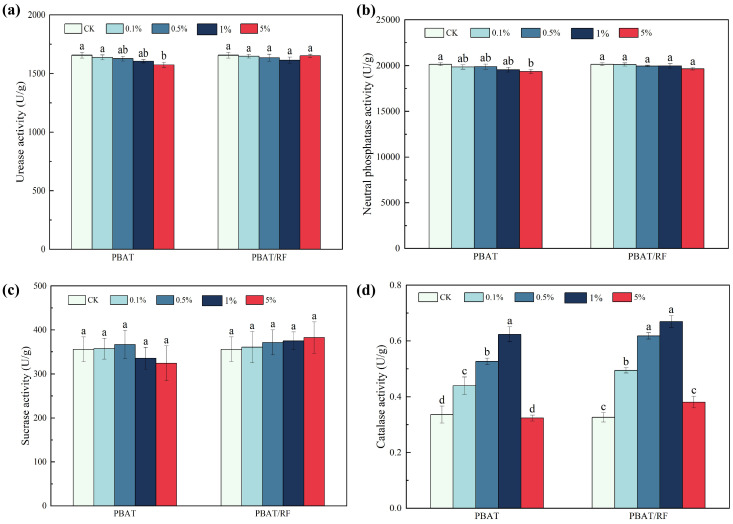
Soil urease (**a**), phosphatase (**b**), sucrase (**c**), and catalase (**d**) activities under the different treatments. The different lowercase letters indicate significant differences across the various treatments at the same growth stage (*p* < 0.05), and the error bars represent the standard deviations.

As illustrated in Figure 8, the soil urease activity peaked in the CK group at 1656.3 U g^−1^, with no significant effects observed for the PBAT and PBAT/RF treatments at any concentration level. Similarly, the soil phosphatase activity reached its highest level in the CK group at 20,156.6 U g^−1^, with no significant impact noted for the PBAT treatments across the various concentrations. In contrast, the soil sucrase activity exhibited an increase with the rise in the PBAT/RF concentration, reaching its peak of 382.8 U g^−1^ at the 5% concentration level. The degradation of the PBAT/RF mulch film, facilitated by the PBAT and reed fibers, contributed to an enhancement in the microbial activity and biomass within the soil, consequently boosting sucrase activity [34]. In comparison to the blank, the soil peroxidase activity exhibited increases at lower PBAT and PBAT/RF concentrations, recording values of 0.44 U g^−1^, 0.53 U g^−1^, 0.63 U g^−1^, 0.49 U g^−1^, 0.62 U g^−1^, and 0.67 U g^−1^ for the PBAT and PBAT/RF at 0.1%, 0.5%, and 1% concentrations, respectively. However, the soil peroxidase activity levels for the PBAT and PBAT/RF at the 5% concentration treatment was 0.32 U g^−1^ and 0.38 U g^−1^, respectively. Soil peroxidase primarily responds to plant and microbial stress under external adverse conditions. The experimental findings indicated that a small amount of residual membrane had a stimulating effect on the soil peroxidase activity [35], while an excessive amount of residual membrane had an inhibitory effect on it.

### 3.4. Effects of the PBAT and PBAT/RF Mulch Films on Soybean Growth

#### 3.4.1. Effects of the PBAT and PBAT/RF Mulch Films on the Germination of the Soybeans

**Figure 9:**
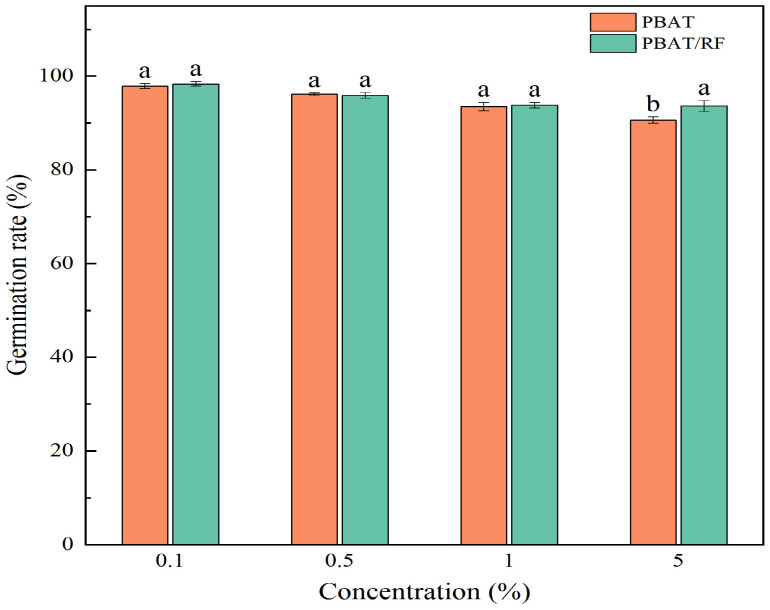
Effects of the PBAT and PBAT/RF on the germination of the soybeans. The different lowercase letters indicate significant differences across the various treatments at the same growth stage (*p* < 0.05), and the error bars represent the standard deviations.

The plant seed germination rate serves as a crucial indicator of a plant’s capacity to sprout under external environmental pressures, reflecting its growth and developmental potential and significantly influencing overall plant growth. In the potting experiment results, the ratio of the germination rates between the experimental group and the blank control group was utilized as the analyzed data, providing valuable insights into the seedling growth performances under the varying conditions. As shown in Figure 9, the germination of the soybeans decreased with increasing concentrations of the mulch fragments in the soil. This inhibitory effect may have stemmed from the hydrophobic nature of the PBAT itself, which disrupted the hydration process of the seed during the critical imbibition stage. The hydrophobicity of the PBAT interfered with the availability of water, thus affecting the ability of the seeds to meet the high hydration requirements needed for successful germination. It was found that an increase in the PE MPs concentration from 0 to 100 mg L^−1^ reduced seed germination from 83.30% to 50.00% [36]. This was similar to the results of this study. In addition, the effect of the PBAT/RF on the germination of the soybean seedlings was less than that of the PBAT with the addition of the same concentration of mulch fragments. As the concentration of the PBAT mulch fragments in the soil increased, soybean germination reached 97.45%, 95.13%, 93.67%, and 90.41%, in that order, while the germination rates of the soybeans as affected by the PBAT/RF mulch fragments were 98.22%, 95.66%, 94.13%, and 93.74%, respectively. The reed fibers in the PBAT/RF had good hydrophilicity, which may have improved the water absorption of the composites.

#### 3.4.2. Effects of the PBAT and PBAT/RF Mulch Films on Soybean Root Length, Plant Height, and Biomass

**Figure 10:**
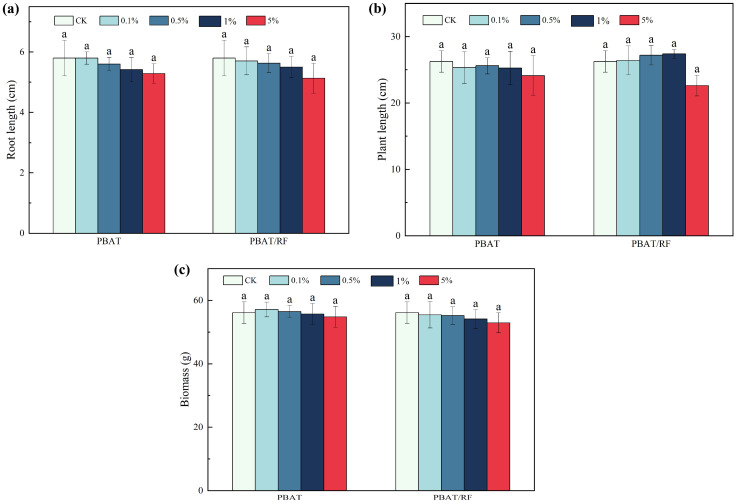
Effects of the PBAT and PBAT/RF on root length (**a**), plant height (**b**), and biomass (**c**) in soybean. The different lowercase letters indicate significant differences across the various treatments at the same growth stage (*p* < 0.05), and the error bars represent the standard deviations.

The data depicted in Figure 10 illustrate the variations in root length, plant height, and total biomass of the soybean seedlings treated with the varying concentrations of PBAT and PBAT/RF mulch film fragments. The lack of substantial effects observed with the different concentrations of the PBAT mulch film fragments on root length, plant height, and total biomass of soybean seedlings may be attributed to the limited degradation of the PBAT mulch film fragments within a brief period. Plants possess internal regulatory mechanisms that enable them to withstand the impacts of external stressors, potentially accounting for the insignificant effects noted. Moreover, the influence of the PBAT fragments on root length, plant height, and total biomass did not demonstrate statistical significance based on the outcomes of the study. The impact of the PBAT/RF mulch film fragments on the growth parameters of the soybean seedlings displayed more variability compared to the PBAT. Notably, the growth indicators exhibited fluctuations in both increases and decreases with the rise in PBAT/RF concentration. When the concentration of the PBAT/RF composite material reached 5%, the root length, plant height, and total biomass of the PBAT/RF decreased by 12.1%, 12.9%, and 6.2%, respectively, compared to the blank control group. This decline was mainly due to the faster biodegradation of the PBAT/RF composite mulch film. At lower concentrations of PBAT/RF in the soil, the reed fibers and PBAT decomposition byproducts acted as nutrients, promoting microbial growth in the soil and enhancing nutrient absorption by the roots. However, an excess concentration led to the production of inhibitory aliphatic and aromatic oligomers and monomers during decomposition, which could impede plant growth, and this was consistent with Li’s study [37].

#### 3.4.3. Effects of the PBAT and PBAT/RF Mulch Films on Plant Chlorophyll

**Figure 11:**
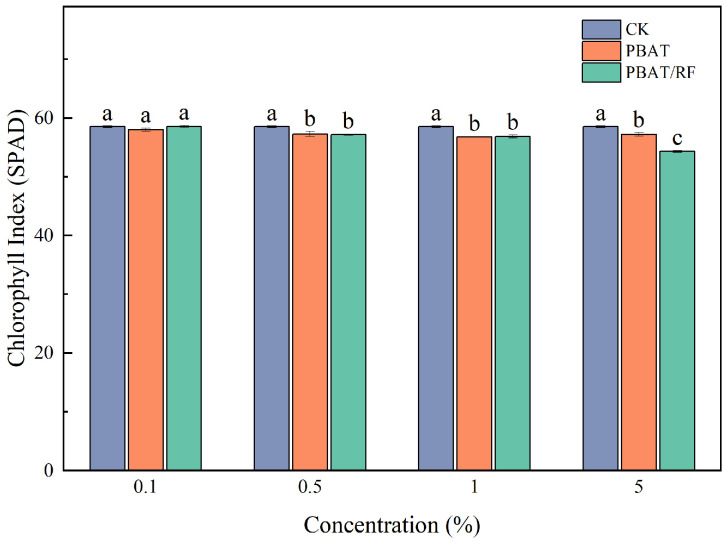
Effect of PBAT and PBAT/RF on the leaf chlorophyll indexes of the soybean seedlings. The different lowercase letters indicate significant differences across the various treatments at the same growth stage (*p* < 0.05), and the error bars represent the standard deviations.

Chlorophyll is an important material basis for plant photosynthesis, and its content effectively reflects the efficiency of a plant in utilizing light energy. The amount of this biosynthesis depends on the presence of chlorophyll in a plant’s leaves, which can be expressed as the SPAD leaf greenness index. The SPAD index is currently considered an excellent indicator expressing the nutritional status of a plant because it is closely correlated with plant yield. In precision agriculture, it is used to determine the demand of a plant for nitrogen fertilization [38]. As shown in Figure 11, the effects of the different concentrations of film fragments on the chlorophyll indexes of the soybean seedling leaves were not significant, which may be due to the natural degradation of the PBAT in the soil environments at slower speeds, and during the experimental period, it may not have been degraded or it may have degraded to a lesser extent. At lower concentrations, the PBAT/RF mulch film fragments had minimal impacts on the chlorophyll indexes of the soybean seedling leaves. However, at a 5% concentration addition, the chlorophyll index decreased by 7.2% compared to the blank control. It should be noted that with the increased PBAT and PBAT/RF doses, the root length, plant length, and biomass decreased, and the urease activity also decreased, which may have indicated changes in the soil’s nitrogen pool. Therefore, the use (biological sorption) of soil nitrogen by the increasing population of soil microorganisms decomposing substrates from the mulch films (especially the PBAT/RF) could have occurred. Therefore, the SPAD indexes in relation to the treatments with high concentrations (5%) of mulch decreased. The chlorophyll content index is closely correlated with the soil nitrogen content and plant yield. Confirmation can be found in Figure 7. The PBAT/RF at 5% caused a significant decrease in the TN content and, at the same time, a significant decrease in the SPAD index (Figure 11). Similar results were found in the study by Wang et al., where PBAT MPs reduced the chlorophyll content of lettuce in a dose-dependent manner, with 0.1 and 1% PBAT-MPs decreasing the chlorophyll content of lettuce by 6.4% and 13.1%, respectively [39].

## 4. Conclusions

After 3 months of composting treatment, the biodegradation rates of the PBAT and PBAT/RF mulch film were 80.91 and 90.43%, respectively, and the biodegradation rate of the PBAT/RF mulch film was significantly higher than that of pure PBAT. The SEM results revealed the formation of larger voids and cracks on the surface of the PBAT/RF film during degradation. These findings indicated that the incorporation of reed fiber into the degradable plastic PBAT enhanced its biodegradation properties and accelerated the degradation rate.

The PBAT mulch film fragments at different concentrations affected the soil properties during the 21-day experimental cycle, with higher concentrations having various levels of effect on both soil properties and soybean growth. The addition of 5% PBAT/RF mulch film fragments decreased the soil pH by 5.1%, increased the soluble organic carbon content by 17.2%, had no significant effect on the soil urease and neutral phosphatase activities, while it increased sucrase activity by 7.4% and decreased catalase activity to 0.38 U/g.

The growth of the soybean seedlings was inhibited, and the chlorophyll contents of the leaves decreased by 7.2%. The effect of a low concentration of residual mulch film on both the plants and soil was weak, but a high concentration of mulch film inhibited plant growth and changed the soil environment accordingly. The germination rate of the soybeans was 93.74% for 5% PBAT/RF, which was 4.29% higher than that of the 5% PBAT treatment group.

At a fragment concentration equivalent to 0.1% of a normal mulch film application, the PBAT/RF mulch film demonstrated a measurable positive impact on the soybean growth parameters, resulting in a 0.77% enhancement in germination rate and a marginal increase in chlorophyll content relative to a conventional PBAT film. These effects were primarily attributable to the accelerated degradation kinetics of the PBAT/RF composite, which exhibited a higher degradation rate compared to standard PBAT formulations. The incorporation of reed fiber (RF) in the composite contributed to this phenomenon by reducing the total PBAT content in the soil matrix and stimulating soil enzymatic activities, particularly sucrase and catalase. Furthermore, the reed fiber component served as a microbial substrate, enhancing both microbial activity and biomass within the soil ecosystem. Observations on N contents in the soil linked with the soil nitrogen and the SPAD index of the leaves indicated the need for nitrogen supplementation in the form of nitrogen fertilization, especially in the cases of higher PBAT/RF concentrations in the soil.

## Figures and Tables

**Table 1:** Compost degradation test protocol.

Project	Type of Sample	Weight of Sample (g)	Weight of Compost (g)
CK	-	-	300
CEL	cellulose	50	300
RF	reed fibers	50	300
PBAT	PBAT	50	300
PBAT/RF	PBAT/RF	50	300

## Data Availability

The original contributions presented in this study are included in the article. Further inquiries can be directed to the corresponding authors.
